# A Formal and Visual Data-Mining Model for Complex Ship Behaviors and Patterns

**DOI:** 10.3390/s22145281

**Published:** 2022-07-14

**Authors:** Yongfeng Suo, Yuxiang Ji, Zhenye Zhang, Jinhai Chen, Christophe Claramunt

**Affiliations:** 1 Navigation College, Jimei University, Xiamen 361021, China; yfsuo@jmu.edu.cn (Y.S.); kyzz6316@gmail.com (Z.Z.); jhchen@jmu.edu.cn (J.C.); 2 Naval Academy Research Institute, 29240 Brest, France; christophe.claramunt@ecole-navale.fr

**Keywords:** complex behavioral pattern, CSBP mining, AIS data, spatiotemporal analysis

## Abstract

The successful emergence of real-time positioning systems in the maritime domain has favored the development of data infrastructures that provide valuable monitoring and decision-aided systems. However, there is still a need for the development of data mining approaches oriented to the detection of specific patterns such as unusual ship behaviors and collision risks. This research introduces a CSBP (complex ship behavioral pattern) mining model aiming at the detection of ship patterns. The modeling approach first integrates ship trajectories from automatic identification system (AIS) historical data, then categorizes different vessels’ navigation behaviors, and introduces a visual-oriented framework to characterize and highlight such patterns. The potential of the model is illustrated by a case study applied to the Jiangsu and Zhejiang waters in China. The results show that the CSBP mining model can highlight complex ships’ behavioral patterns over long periods, thus providing a valuable environment for supporting ship traffic management and preventing maritime accidents.

## 1. Introduction

Maritime transportation has long been a vital mode of international trade, accounting for more than 80% of worldwide exchanges. The continuous increase in maritime traffic and flows has led to critical stakeholder navigation monitoring and security challenges in many overcrowded maritime areas [1]. This entails the need for data mining approaches that can provide decision-aided solutions for extracting unusual and dangerous ship behaviors. Ship behavior pattern mining is an important means to ensure the safety of the navigation environment, but the existing ship behavior pattern mining models are mostly limited to one or several data mining algorithms when conducting trajectory mining research, such as clustering, classification, outlier analysis, or frequent patterns. So, the excavated behavior patterns are not comprehensive enough, not accurate enough, and lack a certain correlation between the behavior patterns; thus, it is difficult to fully grasp the navigation status of ships. In order to improve the situational awareness of maritime supervisors, deter illegal and criminal acts at sea, and maintain the safety of maritime navigation, it is necessary to study the theory and method of situational analysis that can mine the complex behavior patterns of ships. Based on this, this paper introduces a dynamic model in which, first, ship trajectory data is used to summarize ship trajectory characteristics, including temporal and spatial properties. Moreover, ship behaviors are expressed from the macrolevels to the microlevels according to the above characteristics. Secondly, basic ship behaviors are combined by minimum and combination operators to derive complex behaviors, which are mathematically expressed and then visualized. The objective of the modeling approach is to provide a decision-aided system to supervisors when grasping complex ship behaviors, reducing the difficulty of supervision and improving navigation safety.

The rest of the paper is organized as follows. The next section introduces the mathematical modeling of the behavioral and combinatorial patterns. The third section develops the visual component of the model. The fourth section illustrates the potential of the approach through an application example. Finally, the last section summarizes the paper and outlines a few directions for further research.

## 2. Related Work

Previous research oriented toward ship trajectory analysis mainly focused on clustering, classification, outlier analysis, and frequent patterns mining. For instance, Gao et al. [2] proposed a pattern recognition method for the analysis and clustering of ship trajectories and behaviors based on AIS trajectories. Pan et al. [3] introduced a multidimensional trajectory mining and clustering algorithm based on the ship’s type, position, speed, and heading. Zhao et al. [4] applied a DBSCAN algorithm to large AIS trajectory data for characterizing and clustering ship trajectories, which has proven to be successful in determining maritime traffic patterns. Murray et al. [5] developed a specific Gaussian mixture pattern to cluster and classify ship trajectories in a particular sea area. Zhou et al. [6] introduced a ship trajectory pattern analysis whose objective is to reveal complete ship behavioral patterns through changes in ship speed. Zhu et al. [7] incrementally extended the DBSCAN to efficiently classify ship behavioral patterns still at the local level. Wang et al. [8] detected unusual ship behavioral patterns based on K-nearest neighbors (KNN) clustering according to ship attributes and motion context data. Rong et al. [9] introduced a data mining method for probabilistic analysis and anomaly detection of maritime behaviors applied to historical data and based on AIS trajectories and associated data on the Portuguese coast. Karatas et al. [10] extended a Traffic Route Extraction for Anomaly Detection (TREAD) algorithm and applied it to the extraction of trajectories and the detection of unusual patterns. Chen et al. [11] proposed a grid generation method that applied the vertical projection distance and a trajectory frequency pattern mining algorithm based on a vague grid sequence. Shou et al. [12] developed an activity and frequent pattern classification applied to maritime trajectories. Van Hage et al. [13] constructed a simple event model (SEM) for identifying simple motion events, such as stops and movements, and complex motion and event patterns, such as trajectory sequences. Wen et al. [14] introduced a ship behavior semantic model to represent and characterize and annotate specific trajectory patterns. Zhong et al. [15] developed a knowledge-based model of ship behavior according to the COnvention of the internationaL REGulations for preventing collisions at sea (COLREGs) rules. Patroumpas et al. [16] developed a real-time tracking system for the online monitoring of maritime trajectories and the detection of suspicious and dangerous patterns. Such an approach can also provide immediate notification of emergencies, such as suspicious movements in the protected area, to maritime authorities. Huang et al. [17] introduced a topic modeling latent Dirichlet allocation (LDA) to explore the semantic similarity of maritime trajectories.

Despite the interest in the above approach and research, most of the emerging trajectories that have been identified so far do not provide a complete and extensive characterization of the behavioral patterns that encompass all spatial, temporal, and semantic maritime trajectory properties. Most of the existing research on ship behavior patterns has focused on various single mining model algorithms, using clustering, classification, outlier analysis, or frequent patterns to mine the behavior patterns of ships during navigation. However, the single mining model algorithm has some shortcomings, such as the mining ship behavior patterns not being comprehensive and accurate enough to clearly show the correlation between ship behavior patterns. In particular, the identification of complex events and patterns and their associated semantics is still required for many maritime monitoring and prediction activities [18]. To overcome the above-mentioned challenges, this paper combines single behavior patterns to form semantics, and constructs a mining model suitable for complex behavior patterns through visualization and combined with the established similarity model. The goal is to identify frequent and abnormal behavior patterns and discover suspicious ships.

## 3. Ship Behavior and Trajectory Representation

This section formally introduces a representation of ship trajectory and behavioral patterns, and a series of potential combinations of these patterns.

### 3.1. Ship Trajectory Representation

A ship’s behavioral pattern can be derived from its trajectory and changes in speed, course, and location [19]. A ship’s behavior can be revealed by its trajectory and associated semantics. Ship trajectory data derived from AIS samples include the timestamp, longitude, latitude, speed over ground, and course over ground [20]. In addition, each ship is associated to a maritime mobile service identity to distinguish it uniquely [21]. A ship track is composed of track segments and track points. Let us more formally introduce these notions [22]:

**Definition** **1.1.** *(Trajectory Point, P): A ship trajectory point P is represented by a maritime mobile service identification code MMSI, timestamp, longitude, latitude, speed over ground (SOG), and course over ground (COG) P(MMSI) = {Timestamp, Lon, Lat, SOG, COG}*.

**Definition** **1.2.** *(Track Segment, TS): A continuous track segment TS is a subset of a track TR and denoted as follows: TS = {P_i_, P(_i+1)_,…, P_(j−1)_, P_j_}, where i < j, P_(i ≤ m ≤ j)∈ TR*.

**Definition** **1.3.** *(Track, TR): A track TR is a set of track points arranged in chronological order, that is, TR = {P_1_, P_2_,…, P_i_, P_(i+1)_,…, P_n_}, with 0 < I < n*.

### 3.2. Ship Behavior Characteristics

A ship’s behavior should be derived from spatial and temporal properties that characterize a given trajectory explicitly represented by its trajectory properties [23].

#### 3.2.1. Time Characteristics

The temporal characteristics of ship trajectory can be derived by the following properties: time series, series of short time intervals, time persistence, and time interval.

1. Time series: *T* = {$\;t_{a}$,$\;t_{b}$,…,$\;t_{c}$} is a set of timestamps with $\forall t_{m},t_{n}\in T$*,* and *m* < *n*; then, $t_{m}<t_{n}$.
2. A series of short time intervals: *T* = {$\;t_{a}$,$\;t_{b}$,…,$\;t_{c}$} is a time series where any two of its adjacent timestamps $t_{m},\;t_{m+1}\in T$ satisfy $\left|t_{m}-t_{m+1}\right|\geq l$, where g is a time threshold.
3. A series of persistent short time intervals: *T* = {$\;t_{a}$,$\;t_{b}$,…,$\;t_{c}$} is a series of short time intervals with an additional constraint, that is, any of its adjacent timestamps $t_{m},\;t_{m+1}\in T$ satisfy $\left|t_{m}-t_{m+1}\right|\geq l$*,* where *l* is a time threshold.
4. A long interval of time: *T* = {$\;t_{a}$,$\;t_{b}$,…,$\;t_{c}$} denotes the timestamps set of the trajectory of the moving object, and there are two adjacent timestamps *T* in $t_{m},\;t_{m+1}\in T$, *m* ≤ *c − 1*, satisfying $\left|t_{m}-t_{n}\right|\geq h$*,* where *h* is the ss of the long interval of time.

#### 3.2.2. Spatial Characteristics

The spatial characteristics of a ship trajectory mainly include the following definitions which are as follows [24]:

**Definition** **2.1.** *(d-neighborhood): Given a distance threshold d, the d-neighborhood of a trajectory point p is expressed as* $NH_{d}\left(P\right)\;$ *= {q ∈ S│d(p,q) ≤ d}, where d(p,q) denotes the distance between two trajectory points p and q of S the set of trajectory points.*

**Definition** **2.2.** *(Minimum trajectory point core): A minimum trajectory point core mpso(p,MinPts) of a trajectory point p of S is given when its d-neighborhood contains at least MinPts samples, where MinPts denotes a threshold value*.

**Definition** **2.3.** *(d-neighborhood Density): The d-neighborhood density of a trajectory point is given by the number of points of its d-neighborhood*.

**Definition** **2.4.** *(Directly density-reachable): If the trajectory point*$p_{j}$*is situated in the d-neighbourhood of *$p_{i}$*, and *$p_{i}$*is a trajectory point core, *$p_{j}$*is directly obtained from the density of *$p_{i}$.

**Definition** **2.5.** *(Density-reachable): Let us consider the trajectory points*$p_{i}$*,* $p_{j}$*, if there is a sample sequence {*$P_{1}$, $P_{2}\ldots ,\;P_{n}$*}, where*$P_{1}=p1\;and\;P_{n}=pn$*,*$P_{i+1}$*by*$p_{i}$*is called the*$p_{j}$*by*$p_{i}$*density reachable.*

**Definition** **2.6.** *(Density-connected): If objects* $P_{m}$ *, and* $P_{n}$ *are composed of* $P_{k}$ *density values and can be estimated, then* $P_{m}$ *and* $P_{n}$ *are density connected.*

The spatial characteristics of several ship trajectories that can then be studied are as follows [25]:

**Definition** **2.7.** *(Density connectivity): Let us consider moving objects in a sequence of trajectories*$o_{i}$*, and*$o_{j}\in O_{DB}$*, where*$O_{DB}$*is the set of all moving objects. If the trajectory point*$P_{o_{i}}\left(t\right)$*of the moving object*$o_{i}$*is connected with the density*$P_{o_{j}}\left(t\right)$*of the moving object*$o_{j}$*at time t, then we say that*$o_{i}$*and*$o_{j}$*are density connectivity at time t*.

**Definition** **2.8.** *(Spatial proximity): Let P denote a location point and* $o_{i}\in O_{DB}$ *a moving object, if* $P_{o_{i}}\left(t\right)$ *is in the neighborhood of position point P at time t, then we say that the moving object* $o_{i}$ *is in the spatial proximity of target position point P at time t.*

**Definition** **2.9.** *(Positional deviation): Let us consider a moving object*$o_{i}\in O_{DB}$, *if there is a time t of its spatiotemporal trajectory point sequence in which the neighbourhood of its trajectory point is*$NH_{d}\left(P_{o_{i}}\left(t\right)\right)$*and that it is not in the proximity of other moving objects, then we say that the moving object*$o_{i}$*is in positional deviation at time t*.

### 3.3. Ship’s Basic Behaviors

A basic ship behavioral pattern can be expressed as a triple <*N*, *E*, *T*> where *N* denotes an event (i.e., a basic behavior), *E* is the event object, and *T* is the time of the event. We identify a difference between the microlevel where the basic movement of a moving object is analyzed from trajectory data and the macrolevel where moving object trajectories are globally mined.

#### 3.3.1. Microlevel Basic Behavior Patterns

At the microlevel, the basic behavior patterns of ships include the mobile behavior pattern, stay behavior pattern, jumping behavior pattern, and deviation behavior pattern.

(1) Mobile behavior pattern [26] (<Mobile, $o_{i}$, ($t_{a},t_{c}$)>)

An object $o_{i}$ is likely to move at a certain speed, and its trajectory can be either a straight line or a curve composed of a series of spatial points. A mobile behavior pattern is expressed as a sequence of spatiotemporal trajectory points:

(1) $$o_{i}.TR:\;\langle \left(P_{o_{i}}\left(t_{a}\right),t_{a},S_{o_{i}}\left(t_{a}\right),C_{o_{i}}\left(t_{a}\right)\right),\;\left(P_{o_{i}}\left(t_{b}\right),t_{b},S_{o_{i}}\left(t_{b}\right),C_{o_{i}}\left(t_{b}\right)\right),\ldots ,\left(P_{o_{i}}\left(t_{c}\right),t_{c},S_{o_{i}}\left(t_{c}\right),C_{o_{i}}\left(t_{c}\right)\right)\rangle$$

Given a short time interval threshold *g*, a duration threshold *L*, a low-speed movement threshold $\varepsilon_{low}$, and a high-speed movement threshold $\varepsilon_{high}$, the following conditions should be satisfied: (1) time series; (2) short time interval; (3) time duration; and (4) speed stability. The mathematical expression is shown as follows:

(2) $$\left\{ \begin{array}{c} t_{m}<t_{n}\\ |t_{m}-t_{n}|\leq g\\ |t_{e}-t_{d}|\geq L\\ \varepsilon_{low}\leq S_{o_{i}}(t_{m})\leq \varepsilon_{high} \end{array}\right.\begin{array}{cc} \begin{array}{c} \forall t_{m},t_{n}\in T=\{t_{a},t_{b},\ldots t_{c}\},m<n\\ t_{m},t_{m+1}\in T=\{t_{a},t_{b},\ldots t_{c}\},m\leq c-1\\ \forall T^{\prime }=\{t_{d},t_{d+1},\ldots t_{e}\}\subset T=\{t_{a},t_{b},\ldots t_{c}\}\\ o_{i}\in O_{DB},t_{m}\in T^{\prime },\forall T^{\prime }=\{t_{d},t_{d+1},\ldots t_{e}\}\subset T \end{array} & \end{array}$$

(2) Stay behavioral pattern (<STAY, $o_{i}$,($t_{a},t_{c}$)>)

Maritime objects are likely to move at a relatively low speed, thus leading to potential low-speed running areas. A stay pattern highlights a comprehensive behavioral pattern, which not only shows the approximate stillness of motion but also the dense motion of objects in a given area [27]. A stay behavior pattern is expressed as a sequence of spatiotemporal trajectory points:

(3) $$o_{i}.TR:\;\langle \left(P_{o_{i}}\left(t_{a}\right),t_{a},S_{o_{i}}\left(t_{a}\right),C_{o_{i}}\left(t_{a}\right)\right),\left(P_{o_{i}}\left(t_{b}\right),t_{b},S_{o_{i}}\left(t_{b}\right),C_{o_{i}}\left(t_{b}\right)\right),\ldots ,\left(P_{o_{i}}\left(t_{c}\right),t_{c},S_{o_{i}}\left(t_{c}\right),C_{o_{i}}\left(t_{c}\right)\right)\rangle$$

Given the low speed threshold ε low, the following conditions are met: (1) time series; (2) short time interval; (3) time duration; and (4) low speed retention. The mathematical expression is as follows:

(4) $$\left\{ \begin{array}{c} t_{m}<t_{n}\\ |t_{m}-t_{n}|\leq g\\ |t_{e}-t_{d}|\geq L\\ S_{oi}(t_{m})\leq \varepsilon_{low} \end{array}\right.\begin{array}{cc} \begin{array}{c} \forall t_{m},t_{n}\in T=\{t_{a},t_{b},\ldots t_{c}\},m<n\\ t_{m},t_{m+1}\in T=\{t_{a},t_{b},\ldots t_{c}\},m\leq c-1\\ \forall T^{\prime }=\{t_{d},t_{d+1},\ldots t_{e}\}\subset T=\{t_{a},t_{b},\ldots t_{c}\}\\ o_{i}\in O_{DB},t_{m}\in T^{\prime },\forall T^{\prime }=\{t_{d},t_{d+1},\ldots t_{e}\}\subset T \end{array} & \end{array}$$

(3) Jumping behavioral pattern (<Jump, $o_{i}$, ($t_{a},t_{c}$)>)

During the movement of the ship, the AIS equipment is turned off for a long time [28], and a long track point disappears on the track, and the track that is originally a whole segment is divided into two segments. A jumping behavioral pattern is expressed as a sequence of spatiotemporal trajectory points as follows:

(5) $$o_{i}.TR:\;\langle \left(P_{o_{i}}\left(t_{a}\right),t_{a},S_{o_{i}}\left(t_{a}\right),C_{o_{i}}\left(t_{a}\right)\right),\left(P_{o_{i}}\left(t_{b}\right),t_{b},S_{o_{i}}\left(t_{b}\right),C_{o_{i}}\left(t_{b}\right)\right),\ldots ,\left(P_{o_{i}}\left(t_{c}\right),t_{c},S_{o_{i}}\left(t_{c}\right),C_{o_{i}}\left(t_{c}\right)\right)\rangle$$

Given the long-time interval threshold h, the following conditions are met: (1) time series; (2) long-time interval; and (3) time duration. The mathematical expression is shown as follows:

(6) $$\left\{ \begin{array}{c} t_{m}<t_{n}\\ |t_{m}-t_{n}|\geq h\\ {|t_{e}-t_{d}|\geq L}_{} \end{array}\right.\begin{array}{cc} \begin{array}{c} \forall t_{m},t_{n}\in T=\{t_{a},t_{b},\ldots t_{c}\},m<n\\ t_{m},t_{m+1}\in T=\{t_{a},t_{b},\ldots t_{c}\},m\leq c-1\\ \forall T^{\prime }=\{t_{d},t_{d+1},\ldots t_{e}\}\subset T=\{t_{a},t_{b},\ldots t_{c}\} \end{array} & \end{array}$$

(5) Deviation behavioral pattern (<Dev, $o_{i}$, ($t_{a},t_{c}$)>)

A deviation behavioral pattern is defined to convey that a ship motion deviates from most ship motion routes, which is manifested by the distance from the track point of the target object to a typical track exceeding a certain threshold, that is, the track point is outside the main route. The deviation behavioral pattern is expressed as a sequence of position deviation trajectories:

(7) $$o_{i}.TR=\left\{a_{o_{i}}\left(t_{1}\right),a_{o_{i}}\left(t_{2}\right),\ldots ,a_{o_{i}}\left(t_{n}\right)\right\},\;a_{o_{i}}\left(t_{j}\right)=\left(P_{o_{i}}\left(t_{j}\right),t_{j},S_{o_{i}}\left(t_{j}\right),C_{o_{i}}\left(t_{j}\right)\right)\;\;\;$$

Given the quantity scale threshold m, the following conditions are met: (1) time series; (2) short time interval; (3) time duration; and (4) position deviation. The mathematical expression is given as follows:

(8) $$\left\{ \begin{array}{c} t_{m}<t_{n}\\ |t_{m}-t_{n}|\leq g\\ |t_{e}-t_{d}|\geq L\\ Num(P_{o}\in N(o_{i}))\leq m, \end{array}\right.\begin{array}{cc} \begin{array}{c} \forall t_{m},t_{n}\in T=\{t_{a},t_{b},\ldots t_{c}\},m<n\\ t_{m},t_{m+1}\in T=\{t_{a},t_{b},\ldots t_{c}\},m\leq c-1\\ \forall T^{\prime }=\{t_{d},t_{d+1},\ldots t_{e}\}\subset T=\{t_{a},t_{b},\ldots t_{c}\}\\ N(o_{i})\in \{NH_{d}(a_{o_{i}}(t_{1})),NH_{d}(a_{o_{i}}(t_{2})),\ldots ,NH_{d}(a_{o_{i}}(t_{n}))\} \end{array} & \end{array}$$

#### 3.3.2. Macrolevel Basic Behavioral Patterns

At the macrolevel, basic ship behavior patterns can be extracted at the target location of a ship’s arrival, by multi-ship aggregation, and other behaviors [29]. We identify a difference between the gathering and origin destinations and turn back behavioral patterns.

(1) Gather behavioral pattern [30] (<Gather, ($o_{i}$, $o_{j},\ldots$), ($t_{a},t_{c}$)>)

A gather behavioral pattern combines nearby ships from a space-time perspective through density-based clustering, with objects connected by density for a continuous period [31,32] with all ships remaining at low speed. A gather behavioral pattern is represented as a sequence of snapshot clusters, which is composed of multiple objects *o_i_*, *o_j_*,… from a valid snapshot cluster, i.e., <$c_{t_{a}},c_{t_{b}}\ldots ,c_{t_{c}}$>, $C=\left\{c_{t_{a}},c_{t_{b}}\ldots ,c_{t_{c}}\right\}$ is the set of effective snapshot clusters, and *T*= {$\;t_{a}$,$\;t_{b}$,…,$\;t_{c}$} is the timestamp set of effective snapshot clusters, and which meets the following conditions: (1) time series; (2) short time interval; (3) time persistence; (4) density connectivity, and its characteristics are mapped in effective snapshot clusters; (5) mobility stability; and (6) low speed retention. The mathematical expression is shown as follows:

(9) $$\left\{ \begin{array}{c} t_{m}<t_{n}\\ |t_{m}-t_{n}|\leq g\\ |t_{e}-t_{d}|\geq L\\ c_{t_{i}}∼c_{t_{i+1}},\\ S_{c}(t_{m})\leq \varepsilon_{low} \end{array}\right.\begin{array}{cc} \begin{array}{c} \forall t_{m},t_{n}\in T=\{t_{a},t_{b},\ldots t_{c}\},m<n\\ t_{m},t_{m+1}\in T=\{t_{a},t_{b},\ldots t_{c}\},m\leq c-1\\ \forall T^{\prime }=\{t_{d},t_{d+1},\ldots t_{e}\}\subset T=\{t_{a},t_{b},\ldots t_{c}\}\\ c_{t_{i}},c_{t_{i+1}}\in C_{t_{i}}(o_{i})=\{c_{t_{a}},c_{t_{b}},\ldots ,c_{t_{c}}\},t_{i}\in T=\{t_{a},t_{b},\ldots t_{c}\}\\ c\in C_{DB},t_{m}\in T^{\prime },\forall T^{\prime }=\{t_{d},t_{d+1},\ldots t_{e}\}\subset T \end{array} & \end{array}$$

(2) Origin destination behavioral pattern [33] (<OD(place1,place2), $o_{i}$, ($t_{a},t_{b}$)>)

A behavioral pattern of the starting and destination points is considered from a macro perspective. Let *T* = {$\;t_{a}$,$\;t_{b}$} represent the set of time stamps when the moving object reaches the target location, $P_{o_{i}}\left(t_{j}\right)$ represent the position of the moving object $o_{i}$ at $t_{j}$, and the following conditions are met: (1) time series; (2) time interval; and (3) spatial nearest neighbor. The mathematical expression is shown as follows:

(10) $$\left\{ \begin{array}{c} t_{a}<t_{b}\\ |t_{b}-t_{a}|\leq g\\ P_{o_{i}}(t_{j})\in NH_{d}(e_{o_{i}}^{k}(t_{j})) \end{array}\right.\begin{array}{cc} \begin{array}{c} a<b\\ a<b\\ e_{o_{i}}^{m}\in E=\{e_{o_{i}}^{1}(t_{1}),e_{o_{i}}^{2}(t_{2}),\ldots e_{o_{i}}^{m}(t_{n})\},T=\{t_{1},t_{2},\ldots t_{n}\} \end{array} & \end{array}$$

(3) Turn back behavior pattern (<Tback(place1,place2…), $o_{i}$, ($t_{a},t_{q}$)>)

A turn back behavioral pattern is when a ship moving in a given direction is returning in the reverse direction after reaching a given position, and then turns back on the track. The turn back behavioral pattern is expressed as a target position reaching sequence, i.e., $E=\left\{e_{o_{i}}^{1}\left(t_{a}\right),\;e_{o_{i}}^{2}\left(t_{b}\right),\;\ldots ,\;e_{o_{i}}^{m}\left(t_{n}\right),\;e_{o_{i}}^{m-1}\left(t_{n+1}\right),\;\ldots ,e_{o_{i}}^{2}\left(t_{q-1}\right),e_{o_{i}}^{1}\left(t_{q}\right)\right\}$, where $T=\{t_{a}$,$\;t_{b}$, …, $t_{n},\;t_{n+1},\;\ldots ,\;t_{q-1},t_{q}\}$ is the timestamp set of the moving object arriving at the target position, $e_{o_{i}}^{1}\left(t_{a}\right)$ represents the moving object $o_{i}$ arriving at the starting position $e^{1}$ at $t_{a}$, and $P_{o_{i}}\left(t_{j}\right)$ represents the position of the moving object $o_{i}$ at $t_{j}$, meeting the following conditions: (1) time series; (2) time interval; (3) time persistence; and (4) spatial nearest neighbor. The mathematical expression is given as follows:

(11) $$\left\{ \begin{array}{c} t_{m}<t_{n}\\ |t_{m}-t_{n}|\leq g\\ |t_{e}-t_{d}|\geq L\\ P_{o_{i}}(t_{j})\in NH_{d}(e_{o_{i}}^{k}(t_{j})) \end{array}\right.\begin{array}{cc} \begin{array}{c} \forall t_{m},t_{n}\in T=\{t_{a},t_{b},\ldots t_{q}\},m<n\\ t_{m},t_{m+1}\in T=\{t_{a},t_{b},\ldots t_{q}\},m\leq c-1\\ \forall T^{\prime }=\{t_{x},t_{x+1},\ldots t_{y}\}\subset T=\{t_{a},t_{b},\ldots t_{q}\}\\ e_{o_{i}}^{m}\in E=\{e_{o_{i}}^{1}(t_{1}),e_{o_{i}}^{2}(t_{2}),\ldots e_{o_{i}}^{m}(t_{n})\},T=\{t_{1},t_{2},\ldots t_{n}\} \end{array} & \end{array}$$

Table A1 in the Appendix A summarizes the ship’s trajectory characteristics and behavioral patterns.

### 3.4. Behavioral Pattern Combination

As far as basic trajectory patterns are identified at the individual levels, they can be combined to denote behavioral patterns.

#### 3.4.1. Minimum Operation Subset

Combination operations are categorized into logic-based operators, time series-based operators, and other types of operators.

Logic-based operators include logical “and”, “or”, and “none” operations.

Time series-based operator: Time-series relationships are divided into timestamps and interval timestamps operations. As most ship behaviors generally last for a period of time, we consider temporal intervals [34,35]. Thirteen kinds of interval timestamp relationships can be identified according to a temporal algebra. Overall, this identifies a difference between intervals that overlap and do not overlap using different combinations (Table A2 in the Appendix A).

**Figure 1:**
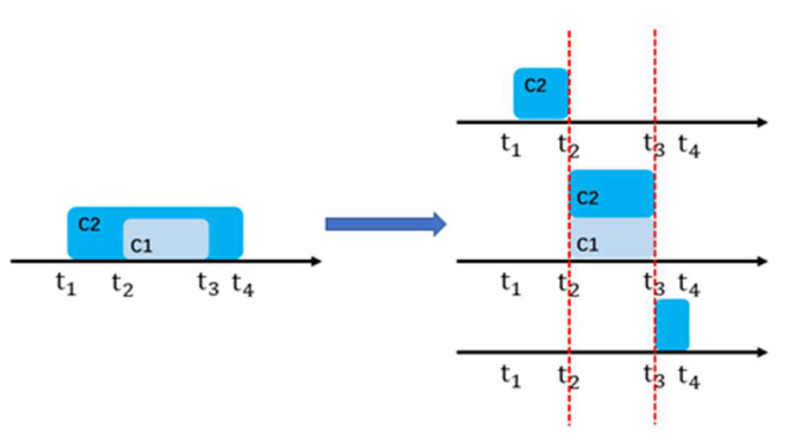
Event time series cutting diagram.

When the interval timestamps of two events do not overlap completely, the sequence of events can be determined. When the interval timestamps of two events overlap completely, the two events are concurrent within the interval timestamp. When the interval timestamps of two events overlap partially, it is difficult to determine whether the events occur in chronological order or whether the events are concurrent, so let us cut the interval timestamps. Figure 1 shows the time series relationship of the two events after cutting. C2 precedes C1 at $t_{1}$–*t*_2_; C1 is concurrent with C2 at $t_{2}–t_{3}$; and C2 follows C1 at $t_{3}$–$t_{4}$. In this case, C1 during C2 is selected for analysis. Other events with partial overlapping time can be cut using this method to determine the sequence of events using similar principles.

Other types of operators are mainly negative operators and time-limited operators, whose semantics are shown in Table 1.

#### 3.4.2. Complex Behavioral Combinatorial Operations

Atomic events are combined based on the minimum operation subset to describe complex ship behavioral patterns, and logical combinatorial operations and temporal combinatorial operations are applied.

A logical combinatorial operation describes complex events through an “and”, “or”, and “non” operations, for example, (*A ∧ B*) *∨ C*.

This example represents events A, B, and C. The following conditions must be met for complex events: events A and B exist, or event C exists.

The sequential combinatorial temporal operations include “before”, “after”, “concurrent”, “negative”, and “time limit”. They are categorized into sequential combinatorial operations, potential sequential combinatorial operations, iterative combinatorial operations, embedded combinatorial operations, and composite operations. The semantics of these combinatorial operations are shown in Table A3 in the Appendix A.

Among these operations, the compound operation is the combination nesting of the above four combination operations: Example: ${\left(A\left(B\gg C\right)-D\right)}_{T}$.

These examples show the following: (1) within the time interval of event A, event B and event C occur successively; and (2) event D will not occur within T after the end of event A.

## 4. Ship Behavioral Pattern (CSBP)—Mining Visual Patterns

### 4.1. Logical Combinatorial Visualization

**Figure 2:**
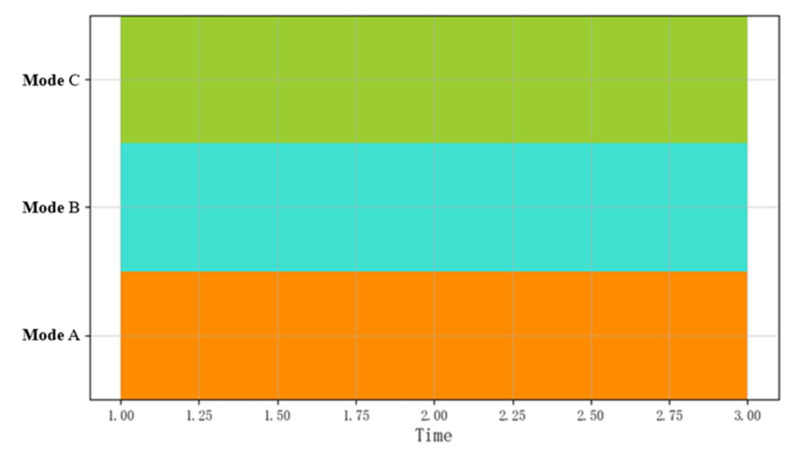
Visual representation of an event flow.

Figure 2 shows the visual representation of a CSBP. The abscissa is time while the ordinate shows different patterns. The different colors in the figure represent different behavior patterns. The logical operator does not consider the time series relationship between the events, so the logical combination operation shown in Figure 2 can be expressed as A∧B∧C.

### 4.2. Temporal Combinatorial Visualization

(1) Sequential combinatorial visualization

**Figure 3:**
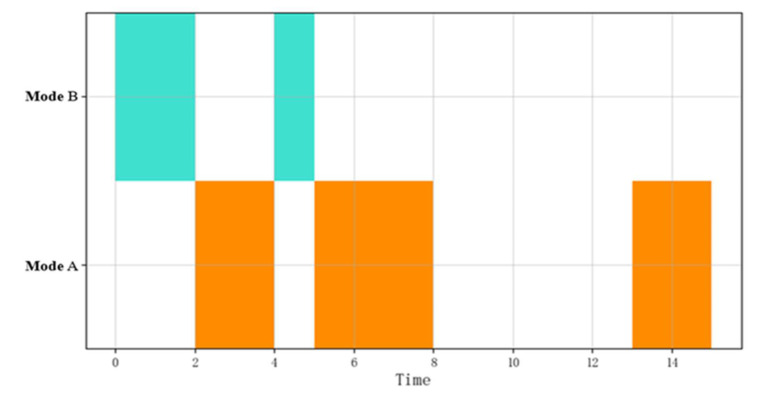
Visualization of the sequential combination of event flows.

Figure 3 shows the visualization diagram of the pattern sequential combination. There is a time series relationship between these patterns, and the sequential combinatorial operation is expressed as B≫A≫B≫A≫A.

(2) Potential sequential combinatorial visualization

**Figure 4:**
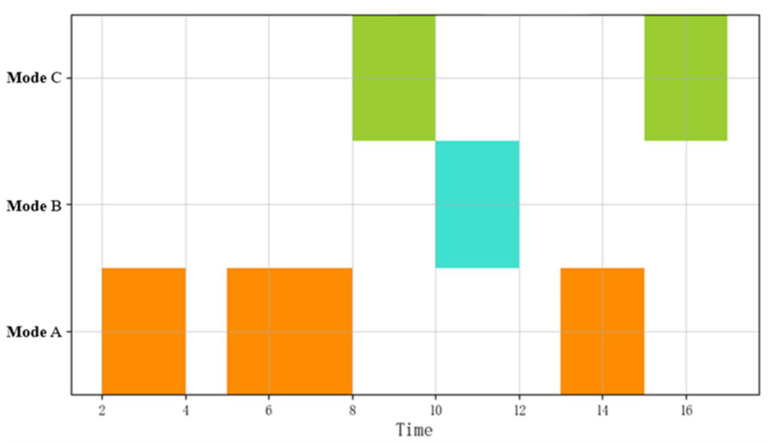
Visualization of the potential sequential combinations of event flows.

Figure 4 shows a visualization of a potential sequence of patterns, which have a time series relationship. Potential sequential combinatorial operations can be expressed as A≫∗≫B≫∗≫C, for unknown events, the symbol “*” is used to indicate, and the patterns of some objects can be omitted to determine the required complex patterns more intuitively.

(3) Iterative combinatorial visualization

**Figure 5:**
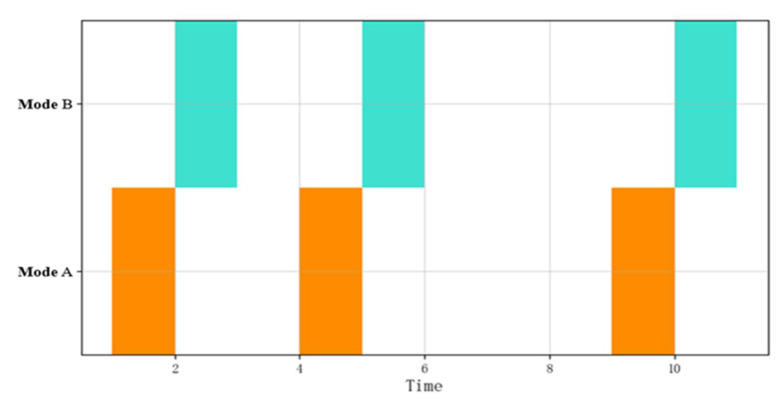
Visualization of the event flow iteration composition.

Figure 5 shows the visual diagram of the pattern iteration combination. From the diagram, patterns A and B can be seen to repeat three times, which can be expressed as $Itr^{3}\left(A\gg B\right)$.

(4) Embedded combinatorial visualization

**Figure 6:**
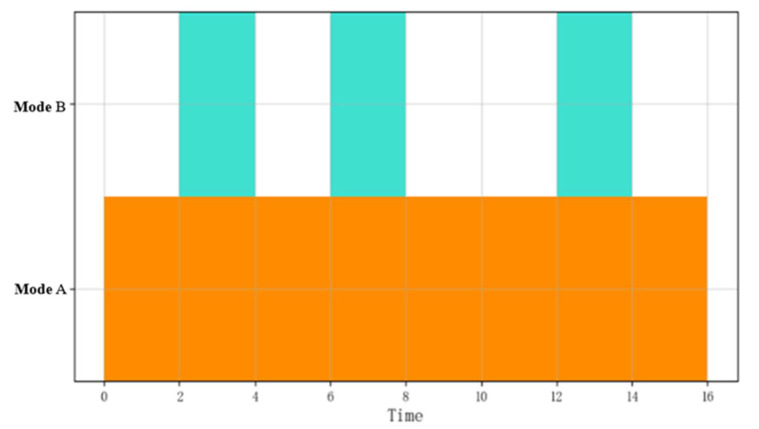
Visualization of the event flow embedded composition.

Figure 6 shows a visual diagram of the pattern embedded combination. From the figure, it can be seen intuitively that during the occurrence of pattern A, pattern B occurs three times in chronological order, which can be expressed as A(B≫B≫B)  or $A\left(Itr^{3}\left(B\right)\right)$.

(5) Composite visualization

**Figure 7:**
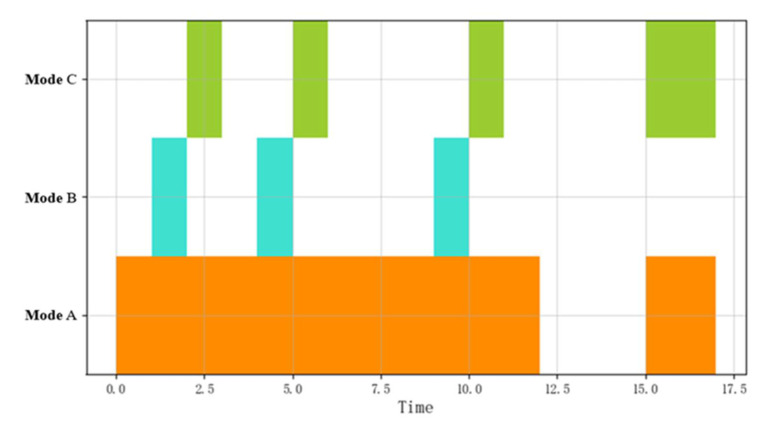
Visualization of the event flow composite composition.

Figure 7 shows a visual diagram of the composite pattern combination. The composite combination combines the above combination operations to form a more complex event, which can be expressed as $A\left(Itr^{3}\left(B\gg C\right)\right)\gg A||C$.

**Figure 8:**
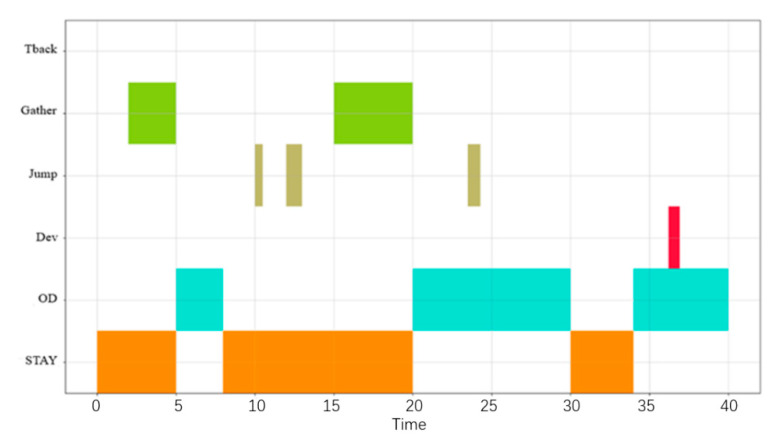
Visualization of a ship CSBP.

Taking the behavioral pattern of a given ship for a given period of time as a case study, the CSBP visual analysis was carried out. Figure 8 shows the visualization diagram of the ships’ CSBP. The abscissa is time and the ordinate is the six behavioral patterns previously described: STAY, OD, DEV, JUMP, Gather, and Tback. The different colors represent different behavioral patterns. The logical combination of operations can be expressed as:

*STAY* ∧ *OD* ∧ *DEV* ∧ *JUMP* ∧ *Gather* (12)

This description indicates that the ship has stayed, then it is at an origin destination, and then a deviation, thus jumping over this period, while there is no turn back behavior pattern. The time series composite operation on the ship can be expressed as:

(13) $$STAY\gg (STAY||Gather)\gg OD\gg \;STAY\left(Itr^{2}\left(JUMP\right)\gg Gather\right)\gg OD\gg \;(OD||JUMP)\gg OD\gg STAY\gg OD\gg (OD||DEV)\gg \;OD$$

The above process shows a ship CSBP in a composite form. This method can accurately and intuitively describe the process of ship CSBP and present in detail complex behavioral patterns during a given navigation.

### 4.3. Visual Analysis of Complex Ship Behaviors

#### 4.3.1. Visualization of a Ship Navigation Behavior

The approach was experimented in the Jiangsu and Zhejiang waters, using a cargo ship as an example, to highlight complex behaviors through the CSBP mining model and used the visual method to illustrate its navigation patterns in 2020. The static information of the cargo ship studied in this experiment is shown in Table 2.

**Figure 9:**
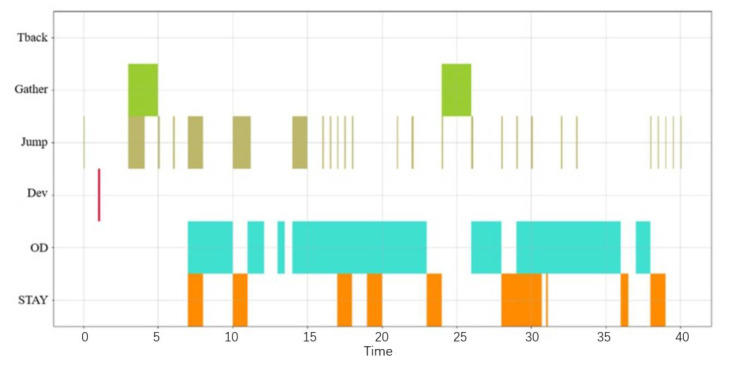
Cargo ship complex behavioral pattern diagram.

Figure 9 shows the visualization of the complex behavior of the cargo during navigation and at different times. The abscissa is UTC (Coordinated Universal Time) time and the ordinate is the five basic ship behavioral patterns previously defined.

#### 4.3.2. Analysis of Complex Ship Behavior

The CSBP mining model can visualize the navigation behavior of a ship during the whole navigation process, which should denote a true reflection of the navigation status of the ship.

**Figure 10:**
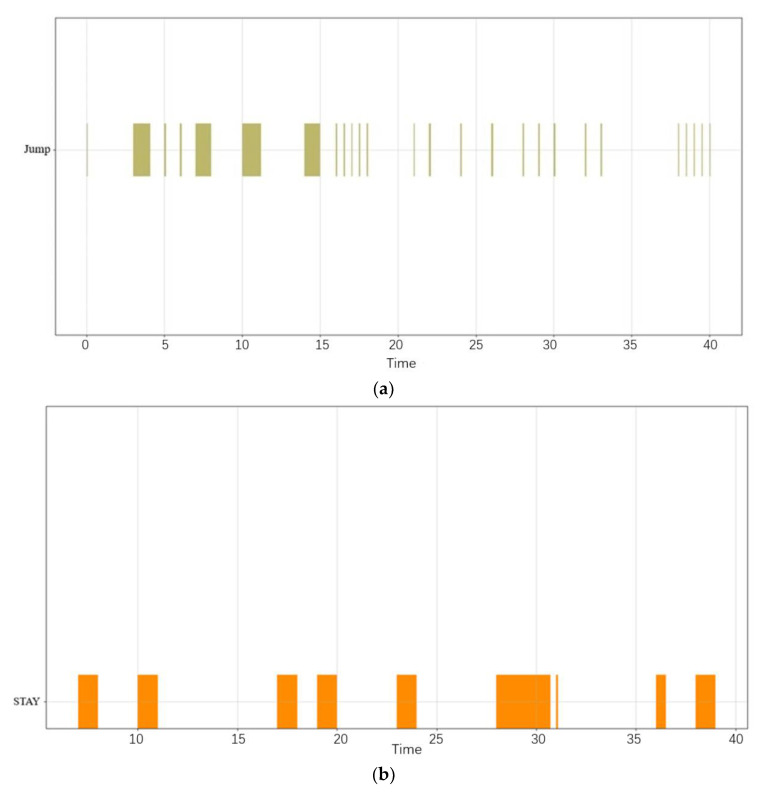
(**a**) The jumping behavior pattern of the cargo ship; (**b**) The stay behavior pattern of the cargo ship.

As shown in Figure 9, the cargo ship showed five behavioral patterns during navigation: stay, origin destination, deviation, jumping, and gather. Regarding the jumping behavioral pattern, this occurred before period 7 and between two adjacent origin destination behaviors, indicating that the cargo ship was in a suspended state and had closed AIS during this period. Several short-time jumping behavioral patterns between the origin destination behaviors indicate that some obstacles may have blocked the AIS signal during the journey of the ship. The ship showed two gather behavioral patterns, and the occurrence time was outside the occurrence period of the origin destination behavior. Therefore, one can infer that the cargo ship could have berthed with the assistance of a tug or overtaken other ships. The ship showed many stay behaviors. One can infer that this location may be an anchor within the period when the behavioral pattern of the origin destination occurred. The speed of the ship was low or zero at this location, and the ship was in the state of anchoring. If the stay behavior was outside the period when the origin destination behavioral pattern occurred, the ship may have berthed at the wharf. From the complex behavioral pattern diagram of the ship, the starting point and arrival point of the cargo ship are clear, no turn back behavior and no other abnormal behavior during navigation was shown, so one can infer that the cargo ship was sailing normally on the planned route. Figure 10a,b are separate representations of the basic behaviors at the microlevel of the cargo ship. Compared with the complex behaviors denoted in Figure 9, the separate jumping behavior and stay behavior lack comparison with the other behaviors and cannot clearly show the state of the ship in time and space, which is not convenient for the overall grasp of the behavior of the ship during navigation.

## 5. Instance Application

The application of the CSBP mining model mining and visualization, combined with a frequent pattern mining algorithm, suspected ships with illegal behaviors might be uncovered.

### 5.1. Visual Analysis of Complex Ship Behaviors

Taking Zhejiang and Fujian waters as the case study, we selected a refined oil smuggling ship. The CSBP mining model visually showed the navigation process of the ship in 2020 and traced the origin of the ship’s smuggling process, combined with the evidence found by the maritime police border guards, which can provide corresponding evidence for the conviction of offenders. The static information of the ships involved in this experimental study is shown in Table 3.

**Figure 11:**
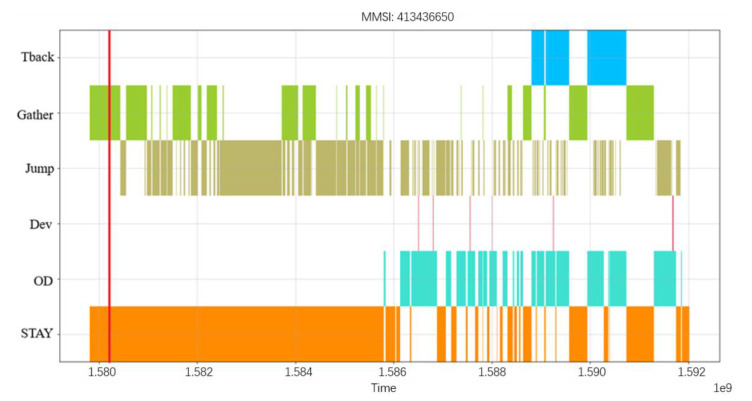
Involved ship complex behavioral pattern diagram.

Figure 11 shows the visualization of the complex ship behavior detected during its navigation, which intuitively shows its behaviors at different times. The abscissa is the UTC of the ship navigation while the ordinate is the six basic ship behavior patterns previously identified. The red straight line represents that the ship passed through stay and gather behavioral patterns at a given time in the navigation process, indicating that two behaviors occurred simultaneously. Shifting the red line from left to right represents the behavioral pattern of the ships in turn over time.

The most prominent suspicious behavior is to stay at a certain place for a long time and frequently turning the AIS system on and off during the stay of the ship. According to the stay behavioral pattern, the ship stayed in the sea area near Zhoushan for nearly 45 days while 81 jumping behavioral patterns occurred during the stay behavioral pattern. This complex behavior pattern can be expressed as $STAY\left(Itr^{81}\left(JUMP\right)\right)$. Accordingly, the characteristics of this suspicious ship are set as follows: (1) the ship showed a stay behavioral pattern, and the stay period was more than 30 days; (2) there were multiple jumps between the behavioral patterns of the ship during this stay period, and the threshold of the number of jumps was set to 10. The judgement basis of suspicious ships chosen above is subjective, which is analyzed here as an example. In practical applications, it will be necessary to combine such patterns with professional knowledge and the relevant evidence of marine police and supervisors to more accurately determine the behavioral characteristics of such suspicious ships.

### 5.2. Frequent Behavior Patterns Uncover Suspicious Ships

**Figure 12:**
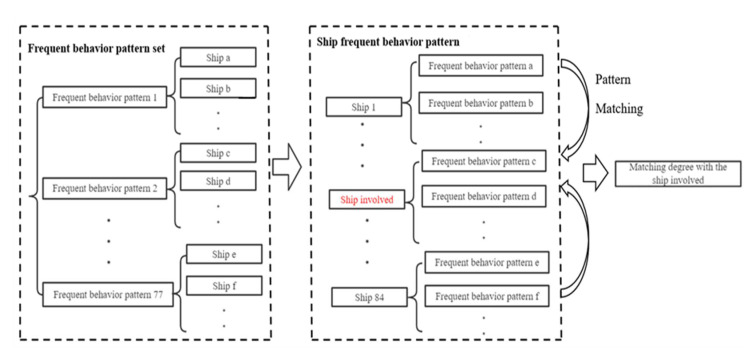
Flow chart of ship frequent behavioral pattern matching.

First, the frequent behavior patterns of a group of ships were mined using the PrefixSpan algorithm. Then, the frequent behavioral pattern was set into the frequent behavior pattern of the ship, the frequent behavioral patterns of other ships were matched with the frequent behavior of the ship involved, and the ships that highly matched with the behavior of the ship involved were uncovered. Figure 12 shows the overall process framework.

The frequent behavioral patterns of the other ships were matched with the frequent behavioral patterns of the ship involved. If the frequent behavior item set of the ship is *S* and the frequent behavior item set of the ship involved is *Q*, the calculation formula of the matching degree is as follows:

(14) $$Match\left(S,\;Q\right)=\frac{\left|S\cap Q\right|}{\left|Q\right|}$$

**Figure 13:**
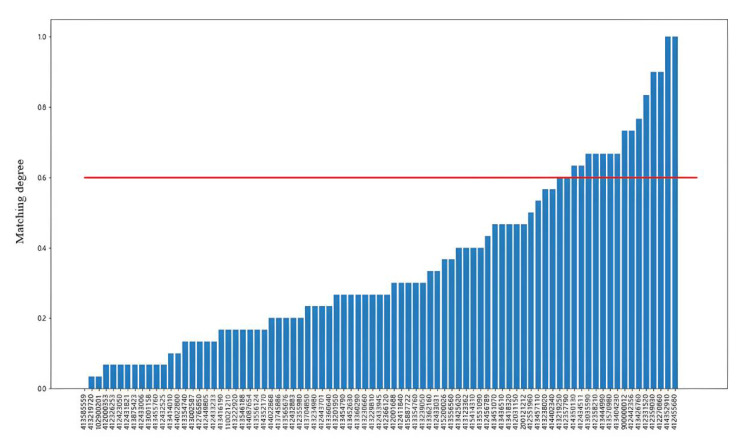
Histogram of the degree of matching of frequent behavioral patterns between the ships and the ship involved.

Figure 13 shows the histogram of the matching degree of the frequent behavior patterns between other ships and the ship involved. Due to the limited cases, the selection of the threshold is relatively subjective. In this case, we selected the threshold M = 0.6. In fact, the selection of the matching threshold could be combined with the actual situation of different cases to achieve the better results. With the increase in the case database, a threshold value is automatically obtained through intelligent algorithm learning, which is also a valuable further research direction.

### 5.3. Uncovering Suspicious Ships

**Figure 14:**
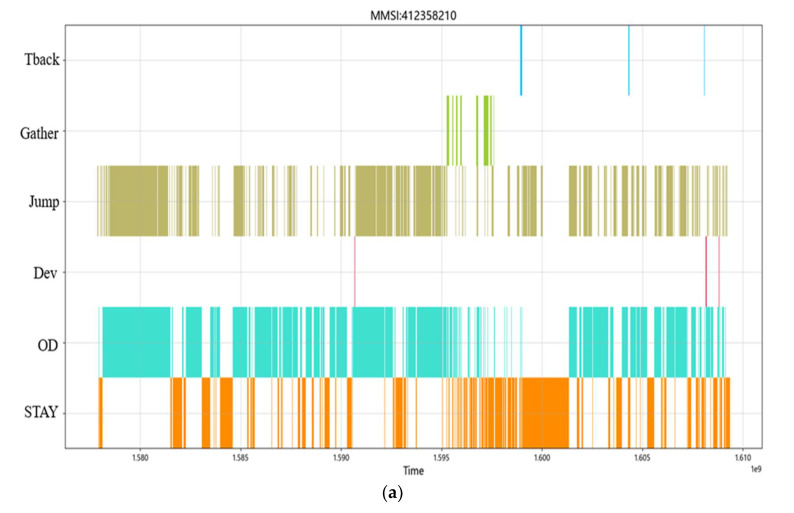

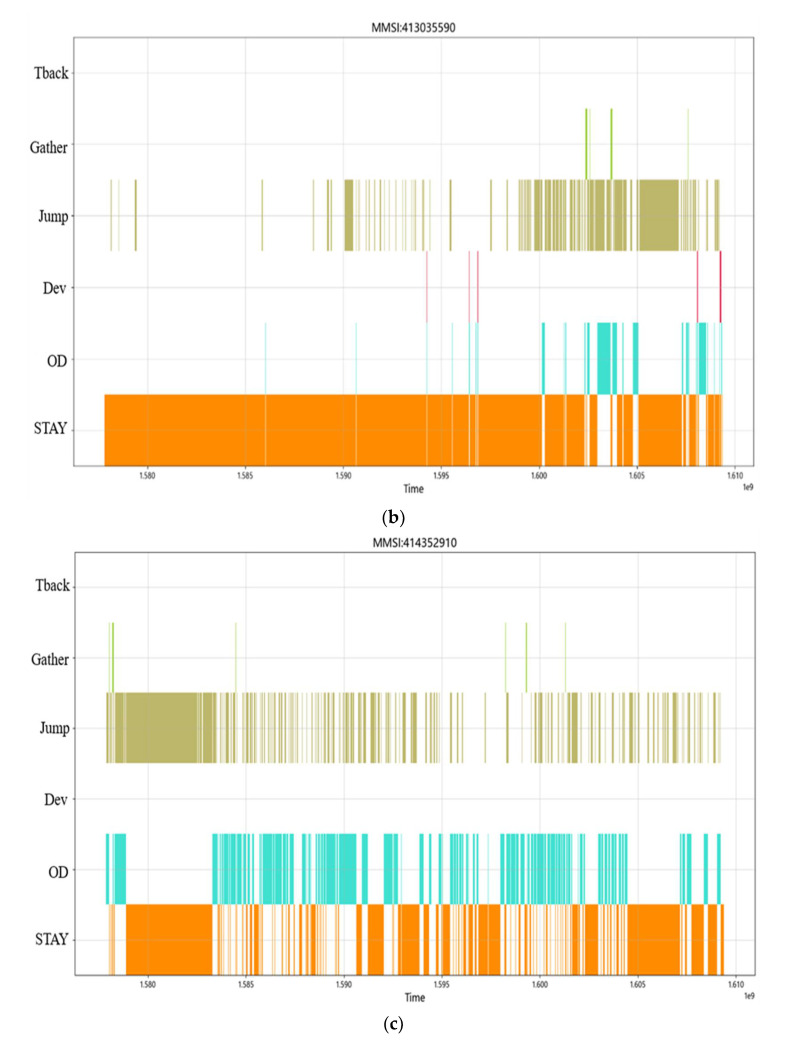
(**a**) Visualization of complex behavior of the first suspicious ship; (**b**) Visualization of complex behavior of the second suspicious ship; (**c**) Visualization of complex behavior of the third suspicious ship.

The mining of the suspected ship requires the illegal and criminal ship behavior as a reference, and the CSBP mining model was used to visually analyze the behavior of the involved ship and uncover the suspicious behavior of the ship. The PrefixSpan algorithm was used to mine the ships closely matching the behavior of the ships involved in the case. Combined with the above two points, suspicious ships can be uncovered. Finally, three ships in this experiment met the above suspicious ship identification requirements, and their visualization results are shown in Figure 14. The complex behavior visualization of the three ships corresponds to Figure 14a–c.

## 6. Discussion and Conclusions

This paper introduces a formal and visual framework for analyzing ship behaviors as derived from AIS trajectory data. Different behavior characteristics are modeled as basic patterns and are combined to denote complex ship behavioral patterns and visualize them using the CSBP mining model. The model method has broad application prospects. For example, by the maritime police, it can be used in the detection of smuggling and drug trafficking. After establishing a certain case database, it can be used to detect illegal activities such as ship smuggling through the judgment of the similarity model. For fishery administration, the corresponding illegal fishing behavior can be identified through the construction of an illegal fishing vessel behavior database and then through the judgment of the similarity model. For the maritime department, it can be used to detect the illegal anchoring and anchor walking behavior. The experimental results show that the CSBP mining model can clearly show ship behavioral patterns during navigation and efficiently uncover suspicious ships. The reliability analysis of the suspected ships uncovered has a higher degree of matching with the involved ship. The aim will be to combine such visual analysis with professional knowledge and maritime law enforcement to derive relevant evidence. A more restrictive study area can be identified before performing frequent behavior pattern mining to reduce the number of ships involved and facilitate the discovery of suspected ships. We also plan to implement the formal and visual principles of the CSBP mining model in a computing architecture and interface that will be specifically designed for maritime officers and experts. Indeed, the approach is still prone to some limitations. First, the quality of the AIS data affects the experimental results. If there is a lot of missing data in the data used in the experiment, some behavior models are difficult to mine, which will affect the analysis of the final experimental results. Secondly, some discontinuous patterns might appear due to AIS signal loss or shade. Last, although six behavioral patterns were identified at the macro- and microlevels, additional patterns might be formally defined; this is left to further work to extend the potential of our whole framework.

## Figures and Tables

**Table 1:** Other operators.

Operator	Expression	Meaning	Temporal Relationship
Negative	${\left(A-B\right)}_{T}$	*B* does not occur in interval *T* after the occurrence of *A*	Time limit condition *T*
Time limit	$A_{T}$	A duration greater than *T*	Time limit condition *T*

**Table 2:** Cargo ship static information table.

MMSI	Ship Name	Ship Type	Ship Length (m)	Ship Width(m)	Draft (dm)
412270860	YUN LI 6	Cargo ship	56	9	3

**Table 3:** Smuggling ship static information table.

MMSI	Ship Name	Ship Type	Ship Length (m)	Ship Width(m)	Draft (dm)
413436650	HX118	Tanker	53	9	36

## Data Availability

The data used to support the findings of this study are available from the corresponding author upon request.

## Appendix A

**Table A1:** Ship trajectory characteristics and behavioral patterns.

	Behavior Patterns	Micro Behavior Patterns				Macro Behavior Patterns		
Behavior Characteristics		Mobile	Stay	Jumping	Deviation	Gather	Origin Destination	Turn Back
Time characteristics	Time series	√	√	√	√	√	√	√
	Short time interval	√	√		√	√	√	√
	Time duration	√	√	√	√	√		√
	Long time interval			√				
Spatial characteristics	Density connectivity					√		
	Spatial nearest neighbor						√	√
	Positional deviation				√			
Other characteristics	Low speed retention		√			√		
	Speed stability	√						
	Mobile stability					√		
	Position sequence symmetry							√

**Table A2:** Interval timestamp relationships.

Category	Category Description	Temporal Relationship	Symbolic Representation	Inverse Notation
The timestamps between the two events do not overlap at all	The sequence of events that can distinguish between two events	C1 before C2	<	>
		C1 meets C2	m	mi
The timestamps between the two events overlap completely	There are two events in a full-time period	C1 equal C2	=	=
The timestamps between the two events overlap	Two events exist at the same time in some time segments	C1 during C2	d	di
		C1 starts C2	s	si
		C1 finishes C2	f	fi
		C1 overlaps C2	o	oi

**Table A3:** Sequential combinatorial operations.

	Definition	Example	Example Semantics
Sequential combination operations	The sequential combinatorial operation requires that events that occur in the target object be sequenced in chronological order.	$A\gg {\left(B\right)}_{T}\gg C\|E$	1. In chronological order, events A, B, and C occur in the order of the event. 2. Event B occurs at least T for a period of time. 3. After event B occurs, event C and event E occur at the same time.
Potential sequential combinatorial operations	The potential sequence combinatorial operation refers to the incomplete confirmation of the sequence of events that occur in the target object, represented by the symbol “*“.	A≫∗≫B	Indicates that the complex event is triggered by the potential sequence of events A and B, and its combination operation expression semantics is as follows: 1. Event A occurs first to the target object in chronological order. 2. Subsequent event B. 3. After event A and before event B, there are none, one or more events.
Embedded combinatorial operation	The embedded combinatorial operation refers to the existence of an event with a large interval, during which other events occur.	A(B≫C≫D)	Indicates that the complex event is composed of four events A, B, C, and D. Among these events, events B, C, and D are embedded in the time interval of event A. The embedded combination operation expresses the semantics as follows: within the time period of event A, event B, event C, and event D occur successively.
Iterative combinatorial operation	The iterative combinatorial operation refers to the events that occur in the target object, which occur iteratively in the time series, represented by $Itr^{n}\left(*\right)$.	$Itr^{n}(A\gg B\gg C)$	1. Event A, event B, and event C occur in chronological order. 2. The whole process is iterated *n* times.
